# ATM and P53 differentially regulate pancreatic beta cell survival in Ins1E cells

**DOI:** 10.1371/journal.pone.0237669

**Published:** 2020-08-18

**Authors:** Celina Uhlemeyer, Nadine Müller, Kerstin Grieß, Corinna Wessel, Caroline Schlegel, Jennifer Kuboth, Bengt-Frederik Belgardt

**Affiliations:** 1 Institute for Vascular and Islet Cell Biology, German Diabetes Center, Leibniz Center for Diabetes Research at Heinrich Heine University, Düsseldorf, Germany; 2 German Center for Diabetes Research (DZD e.V.), Neuherberg, Germany; University of Michigan, UNITED STATES

## Abstract

Pancreatic beta cell death is a hallmark of type 1 and 2 diabetes (T1D/T2D), but the underlying molecular mechanisms are incompletely understood. Key proteins of the DNA damage response (DDR), including tumor protein P53 (P53, also known as TP53 or TRP53 in rodents) and Ataxia Telangiectasia Mutated (ATM), a kinase known to act upstream of P53, have been associated with T2D. Here we test and compare the effect of ATM and P53 ablation on beta cell survival in the rat beta cell line Ins1E. We demonstrate that ATM and P53 differentially regulate beta cell apoptosis induced upon fundamentally different types of diabetogenic beta cell stress, including DNA damage, inflammation, lipotoxicity and endoplasmic reticulum (ER) stress. DNA damage induced apoptosis by treatment with the commonly used diabetogenic agent streptozotocin (STZ) is regulated by both ATM and P53. We show that ATM is a key STZ induced activator of P53 and that amelioration of STZ induced cell death by inhibition of ATM mainly depends on P53. While both P53 and ATM control lipotoxic beta cell apoptosis, ATM but not P53 fails to alter inflammatory beta cell death. In contrast, tunicamycin induced (ER stress associated) apoptosis is further increased by ATM knockdown or inhibition, but not by P53 knockdown. Our results reveal differential roles for P53 and ATM in beta cell survival *in vitro* in the context of four key pathophysiological types of diabetogenic beta cell stress, and indicate that ATM can use P53 independent signaling pathways to modify beta cell survival, dependent on the cellular insult.

## Introduction

Insulin secretion from pancreatic beta cells is essential for mammalian life. Hence, a loss of beta cell function leads to the metabolic disease diabetes mellitus. While type 1 diabetes (T1D) is characterized by massive inflammatory beta cell death, the more prevalent form, type 2 diabetes (T2D) is usually associated with obesity and characterized by reduced insulin secretion, eventually followed by a partial loss of beta cell mass due to beta cell death and potentially dedifferentiation [1–7]. In detail, cell death and moderate to near complete loss of beta cells is detectable in multiple genetic and pharmacological animal models of T1D and T2D [8–12]. Similar findings have been described in studies of T1D or T2D donor pancreata, although there is clear patient to patient variation [2–7]. Programmed cell death (apoptosis) of beta cells also reduces the efficacy of islet transplantation preparations [13] and is a physiological process after pregnancy, at least in mice [14]. Several signaling pathways and events of cell stress have been linked to insufficient insulin secretion and beta cell death in T2D. These include an increase in unfolded proteins in the endoplasmic reticulum (ER), termed ER stress [15], oxidative stress and DNA damage [8, 16], cytokine induced inflammatory signaling [17], as well as induction of cell death upon hyperlipidemia (known as lipotoxicity) [18]. Other mechanisms, including an imbalance of cellular microRNAs [8, 19] or aggregation of amyloid [20], have also been shown to be involved in beta cell demise.

Apoptosis is a complex molecular process that needs to be under strict cellular control at all times. The arguably best studied regulator of apoptosis is the transcription factor tumor protein P53 (P53, known as transformation related protein TP53, or TRP53 in rodents), typically associated with the DNA damage response (DDR). Multiple proteins regulate P53 through post-translational modifications, which allows to couple the general cellular state to P53 activity. In turn, P53 can stimulate DNA repair, block proliferation, effect senescence, and induce apoptosis, according to the severity and duration of the specific cell stress, including, but not limited to, DNA damage [21]. Regarding beta cells, we previously linked P53 activation to prediabetic upregulation of the microRNA-200 family in mouse models for beta cell apoptosis and T2D [8].

P53 stability, localization and activity is regulated by many post-translational modifications [22]. Hence, the kinase Ataxia Telangiectasia Mutated (ATM) mediates DNA damage induced activation of P53 and belongs to the DNA damage response (DDR). Notably, patients with ATM loss of function mutations have a higher incidence of (obesity-independent) diabetes [23]. On the other hand, ATM activation was detected in beta cells of T1D patients, and in a recent report, beta cell specific ATM ablation protected against chemically induced beta cell death in mice [24]. While ATM can regulate P53 activity via phosphorylation, recent investigations have focused on either P53 or ATM, but not if both act additively, synergistically or independent of each other. In turn, it is unknown if ATM and P53 activation by different types of diabetogenic stress regulate beta cell demise in the same manner. Since DDR activation is a hallmark of beta cells in T2D patients [16, 25], we set out to assess if manipulation of ATM mirrors the effect of P53 ablation, and would be sufficient to ameliorate beta cell death induced by four main diabetogenic stress types in Ins1E cells.

## Material and methods

### Cell culture and transfection

Ins1E cells [26] were cultivated in RPMI 1640 with 10% FCS, 2 mM glutamax, 1 mM sodium pyruvate, 11.2 mM HEPES, 0.175 mM beta-mercaptoethanol and 100 U/ml penicillin/streptomycin. Cells were routinely checked for mycoplasma infection and their identity confirmed by qPCR, western blots and immunostainings for beta cell marker mRNAs and proteins, respectively. All transfections were performed using Lipofectamine RNAiMax (13778, Life Technologies), according to the product manual and with a final concentration of 50 nM (either 50 nM of a single used siRNA or 2 x 25 nM in combination treatments in Fig 6). SiRNA sequences were synthetized by Eurogentec (if not further mentioned) and were as follows: si-CTRL (pool of 5’-GCA-GCA-CGA-CUU-CUU-CAA-GTT-3’ and AGG-UAG-UGU-AAU-CGC-CUU-GTT) [8], si-P53 (GAA-GAA-AAU-UUC-CGC-AAA-ATT), si-ATM (GGU-CUA-CGA-UAC-UCU-UAA-ATT). Cells were harvested 30–72 h post transfection.

### Chemicals and diabetogenic treatments

Ins1E cells were treated with: DMSO (D8418, Sigma-Aldrich), Nutlin-3 (10 μM for 24 h, N6287, Sigma Aldrich), tunicamycin (Tunica, 2 or 6 μg/ml for 6, 24 or 48 h, T7765, Sigma-Aldrich), thapsigargin (Thapsi, 1 μM for 6 h, T9033, Sigma), KU-60019 (KU, 0.1 to 1 μM for 6, 16, 24 or 48 h treatment with always 3 h pre-treatment, SML1416, Sigma-Aldrich), essentially fatty acid free bovine serum albumine (BSA, A8806, Sigma-Aldrich), sodium palmitate (Palm, 500 μM for 24 h, P9767, Sigma-Aldrich), a cytokine mix (Cyto) composed of tumor necrosis factor alpha (TNFα, 1000 U/ml for 24 or 48 h, Prc3014 or Phc3015, Thermo Fisher), interleukin 1 beta (IL1β, 50 U/ml for 24 or 48 h, Phc0814, Thermo Fisher), interferon gamma (IFNγ, 767 U/ml for 24 or 48 h, Phc4031, Thermo Fisher), or streptozotocin (STZ, 1.5 mM for 4, 6, 8, 16 or 24 h, S0130, Sigma-Aldrich). Due to its established instability in solution, STZ powder was kept at -20°C at all times and was always diluted (in full Ins1E medium) immediately before treatment. In case of deviating concentrations used, the exact concentration is stated in the figure legends.

### Immunoblotting

Ins1E cells were treated in 6-well plates according to the following schemes. In case of siRNA mediated knockdown: 3 wells were transfected with control siRNA and 3 wells with siRNA against the mRNA of interest (P53/ATM). 2 wells of each were treated with the various small molecules as technical duplicates, if only one treatment condition was tested (16 h STZ, 24 h Cyto, Palm and Tunica) and as single well if two treatment conditions were tested (4 h and 8 h STZ or 6 h Tunica and Thapsi), and one well always with diluent as unstressed control. In case of KU treatment: 3 wells were treated with DMSO and 3 wells with 1 μM KU for two treatment conditions (4 h and 8 h STZ or 6 h Tunica and Thapsi) or 2 wells each with DMSO, 0.1 and 1 μM KU if only one treatment condition was tested (16 h STZ). Supernatant (containing dead/dying cells) and trypsinized cells were spun down for 5 min at 300*g, washed twice with PBS and the resulting cell pellet lysed in RIPA buffer (ab156034, Abcam) with cOmplete Mini Protease Inhibitor Cocktail and PhosStop (both from Sigma-Aldrich) or in Bio-Plex buffer (171304011, Bio-Rad). Protein concentrations were measured using the Bicinchonic Acid (BCA) method. 4x Laemmli buffer (1610747, Bio-Rad) was added to samples, approx. 20 μg protein samples were separated by SDS-PAGE using self-cast (4% stacking and 12% separating gel) or pre-cast 4–15% Stainfree gels (4568086, Bio-Rad), transferred by electroblotting and membranes blocked in TBS with 0.1% Tween-20 and 5% nonfat-dried milk or BSA for 1 h. Membranes were incubated with appropriate antibodies at 1:1000 overnight at 4°C, washed thrice, exposed to HRP-conjugated or fluorescent labelled secondary antibodies for 1 h at room temperature and developed using ECL Western Blotting Substrate (170–5061, Bio-Rad). Beta ACTIN, alpha TUBULIN or total protein amount detected by using a proprietary trihalo compound to enhance the fluorescence of tryptophan amino acids when exposed to UV light (contained in pre-cast Stainfree gels) were used for normalization of all bands of interest. Quantification of all bands was performed using Image Lab software (Bio-Rad). For quantification of technical replicates, the value of the band of interest of the first small molecule treated control siRNA well was set to 1, and all other values normalized to that. Values of technical replicates were averaged for each experiment. Uncropped membranes are depicted in S5 Fig.

### Flow cytometric cell death analysis

Ins1E cells were treated in 24-well plates as a single technical replicate, based on minimal interexperimental variability experienced. For Fig 6, cells were treated according to the following scheme: 4 wells each were treated with control siRNA, si-P53, si-ATM, KU, si-P53+si-ATM or si-P53+KU. The amount of DMSO and siRNA was adjusted in all wells using pure DMSO and control siRNA. All treatments were started simultaneously 24 h post transfection and adapted for significant cell death detection regarding concentration (Tunica, 6 μg/ml) and treatment durations (Cyto and Tunica, 48 h). After 24 to 48 h treatment, the supernatant (containing dead/dying cells) and trypsinized cells were spun down for 5 min at 300*g and the resulting cell pellet was resuspended in PBS containing propidium iodide (PI, 1:500, P3566, Thermo Fisher). After 10 min incubation, the amount of PI positive and negative cells was measured using BD FACSCalibur^TM^. Percentage of living (PI negative) cells was quantified using FlowJo V10 software (BD Biosciences).

### Flow cytometric EdU assay

Ins1E cells were treated in 12-well plates. The EdU experiments were performed in technical duplicates or triplicates. Cells were transfected with control siRNA or siRNA targeting P53. 48 h after transfection, the cells were treated with 5 μM EdU and 1 well with medium (negative control). After 2.5 h of EdU incorporation, supernatant and trypsinized cells were harvested as described above and the EdU assay was performed according to manufacturer’s instructions (C10425, Thermo Fisher). The amount of EdU positive and negative cells was measured using BD FACSCalibur^TM^. Percentage of EdU positive cells was quantified using FlowJo V10 software (BD Biosciences).

### RNA isolation and quantification

Ins1E cells were either treated in 24-well plates (Fig 6A, in technical quadruplicates), 12-well plates (S1 Fig and S2A Fig–S2E Fig, in triplicates) or in 6-well plates (S3A Fig, in triplicates). RNA was isolated using Trizol reagent (30–2020, VWR) according to the manufacturer’s protocol. RNA was reverse transcribed using High-Capacity cDNA Reverse Transcription Kit (4368813, Thermo Fisher). Quantitative realtime PCR was performed in a Quantstudio 7 (Applied Biosystems) by using gene specific primers (S1 Table) and Perfecta Sybr Green FastMix (733–1391, VWR). Ct values were normalized to *36B4*, or *36B4* and *GUSB* mRNA levels, as stated in the figure legends.

### Antibodies

The following antibodies were used: alpha TUBULIN (ab89984, Abcam); ATF4 (11815, Cell Signaling); BAX (14796, Cell Signaling); beta ACTIN (sc-47778, Santa Cruz); CASPASE 3 (14220, Cell Signaling), cleaved CASPASE 3 (9661, Cell Signaling); (cleaved) CASPASE 9 (9508, Cell Signaling); cleaved PARP1 (ab32064, Abcam); IκBα (4812, Cell Signaling); P21 (ab109199, Abcam); p-ATM/ATR Substrate Motif (6966, Cell Signaling); PDX1 (5679, Cell Signaling); p-IRE1α (NB1002323, Novusbio); pS139-H2A.X (9718, Cell Signaling); p-P38 MAPK (Thr180/Tyr182; 4511, Cell Signaling); pS15-P53 (12571, Cell Signaling); PUMA (14570, Cell Signaling) and XBP1s (12782, Cell Signaling).

### Automated Ins1E cell counts

Staining and quantification of Ins1E cells was performed as published [8] with the modification that imaging was performed at a confocal microscope (LSM 880, Zeiss).

### Statistical analysis

Values are reported as average ± s.d. showing each individual experiment as a single data point. The number of replications (independent experiments) is stated in each figure legend and also visible in the quantification figures. No statistical method was used to predetermine sample size, but instead was based on preliminary data and previous publications as well as observed effect sizes. Due to the small sample sizes, normality testing was not performed. Each statistical test is described in the figure legends. Depending on the number of variables, a regular one- or two-way analysis of variance (ANOVA), followed by Sidak’s multiple comparison test was used, comparing all conditions against each other. Experiments with only two groups being tested were analyzed by an unpaired two-sided Student’s t-test. Statistical analyses were performed using the Graphpad Prism (GraphPad Software, La Jolla, CA, USA, Version 7) software. P-values are shown in each figure and are rounded to the third decimal place.

## Results

### Both ATM and P53 mediate stress signaling and beta cell apoptosis upon DNA damage

**Fig 1:**
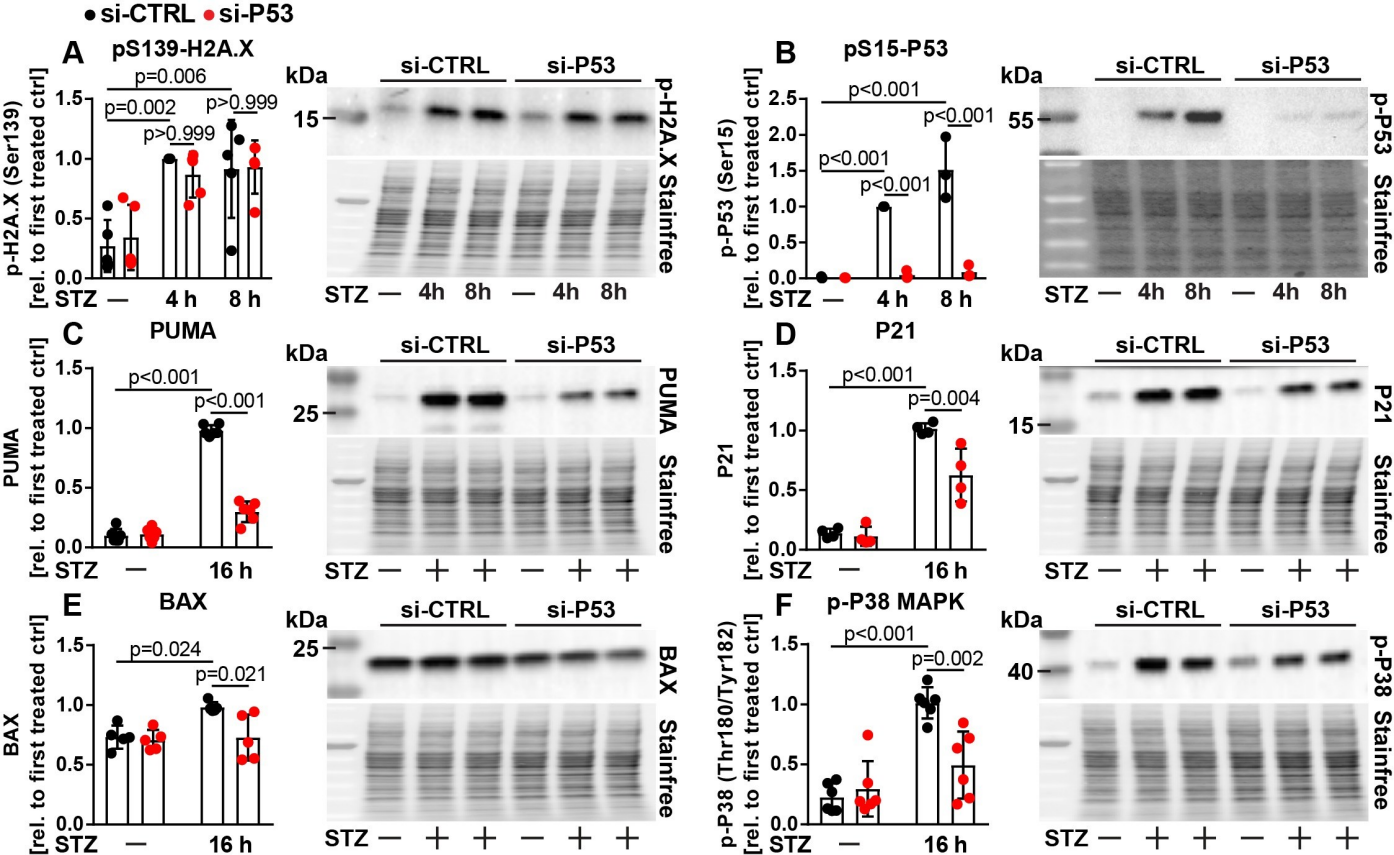
P53 modulates STZ induced stress signaling pathways in Ins1E cells. Relative protein amount of (A) pS139-H2A.X, (B) pS15-P53, (C) PUMA, (D) P21, (E) BAX and (F) p-P38 MAPK in Ins1E cells transfected with control siRNA or siRNA targeting P53. 48 h post transfection, cells were treated for (A+B) 4 h or 8 h or (C-F) 16 h with STZ or medium as control (n = 3–6 independent experiments and one representative immunoblot). The total protein content was used as loading control. The first treated control was set to 1. Significance was determined by two-way ANOVA followed by Sidak’s multiple comparison test.

We investigated the role of ATM and P53 in the commonly used rat immortal beta cell line Ins1E. Ins1E cells were generated by irradiation, show robust glucose stimulated insulin secretion over many passages, and recapitulate key aspects of beta cell physiology and apoptosis in T2D such as sensitivity to DNA damage, inflammation, lipotoxicity and ER stress [8, 9, 26–28]. While the mouse beta cell line Min6 is also often used to study beta cell physiology *in vitro*, it was generated by overexpression of the established P53 inhibitor Simian Virus 40 (SV40) T-Antigen, hence we limited our studies to Ins1E cells in this report [29]. To verify that P53 signaling is wildtype-like in (our clonal population of) Ins1E cells, we confirmed that pharmacological activation of P53 (with the small molecule Nutlin-3 [30]) induces activation of its canonical target genes including *MDM2* [21] and *PHLDA3* [31], which was revertable upon siRNA mediated knockdown of P53 (S1A Fig–S1D Fig). Mechanistically, Nutlin-3 indirectly induces stabilization and activation of P53, which simulates increased P53 signaling found in beta cells of patients with T2D [16]. Treatment with Nutlin-3 also induced mRNA expression of the P53 modulator *MDM4* [32], but not of *PDX1*, a key beta cell transcription factor [33] (S1C Fig and S1E Fig). Moreover, phosphorylation of P53 on serine 15 (pS15-P53) was stimulated by Nutlin-3 incubation, which is important for activation of P53 [21, 34] (S1F Fig). In line with this, Nutlin-3 induced apoptosis in Ins1E cells only in presence of P53, as shown by immunoblotting for cleaved CASPASE 3 (CC3), an established marker for apoptosis [35] (S1F Fig). Since one key role of P53 is to limit proliferation, we next assessed proliferation of Ins1E cells 48 h after transfection with siRNA targeting P53 or control siRNA. Knockdown of P53 increased proliferation of Ins1E cells as determined by automated cell counts as well as incorporation of the artificial nucleotide 5-ethynyl-2'-deoxyuridine (EdU) [36], further indicating that Ins1E cells demonstrate functional P53 signaling (S1G Fig and S1H Fig). To gain mechanistic insights into how ATM and P53 interact and regulate beta cell death, we first performed streptozotocin (STZ) treatments with or without P53 knockdown, followed by detection of multiple intracellular signaling events by immunoblotting. STZ is a commonly used chemical that enters beta cells selectively through the glucose transporter GLUT2 and induces oxidative stress and DNA damage, recapitulating similar events detected in beta cells of human T2D patients [25]. Similar to Nutlin-3 treatment, short term treatment of Ins1E cells with STZ (6 h) [8, 37–40] induced mRNA expression of *MDM2*, *MDM4*, as well as *PHLDA3*, which was blunted by P53 knockdown (S2A Fig–S2D Fig). In contrast, expression of *PDX1* was reduced by STZ treatment, but not additionally modulated by P53 knockdown, demonstrating the specificity of the observed signaling events (S2E Fig). Experiments with 4 h and 8 h STZ treatment revealed that STZ induces the DNA damage response and P53 activation, as determined by phosphorylation of histone H2A.X at serine 139, a marker for DNA double strand breaks (Fig 1A) [41], and an increase in pS15-P53 (Fig 1B). P53 knockdown did not alter phosphorylation of H2A.X, but (as expected) almost completely abolished detectable pS15-P53 (Fig 1A and 1B). In contrast, neither STZ treatment nor P53 knockdown altered levels of IĸBα, a protein involved in inflammatory signaling (S2F Fig). We next assessed, if STZ mediated activation of P53 would eventually result in an increase in canonical P53 target proteins. Indeed, 16 h treatment with STZ elevated cellular levels of the proapoptotic protein PUMA (also known as BBC3), the cell cycle regulator P21 (also known as CDKN1A) and (to a lesser extent) the apoptotic regulator protein BAX, all of which were reduced by P53 knockdown (Fig 1C–1E). Notably, knockdown of P53 also partially prevented STZ induced activation (phosphorylation) of the stress associated P38 mitogen activated protein kinase (P38 MAPK), but did not affect PDX1 expression at that time point (Fig 1F and S2G Fig).

**Fig 2:**
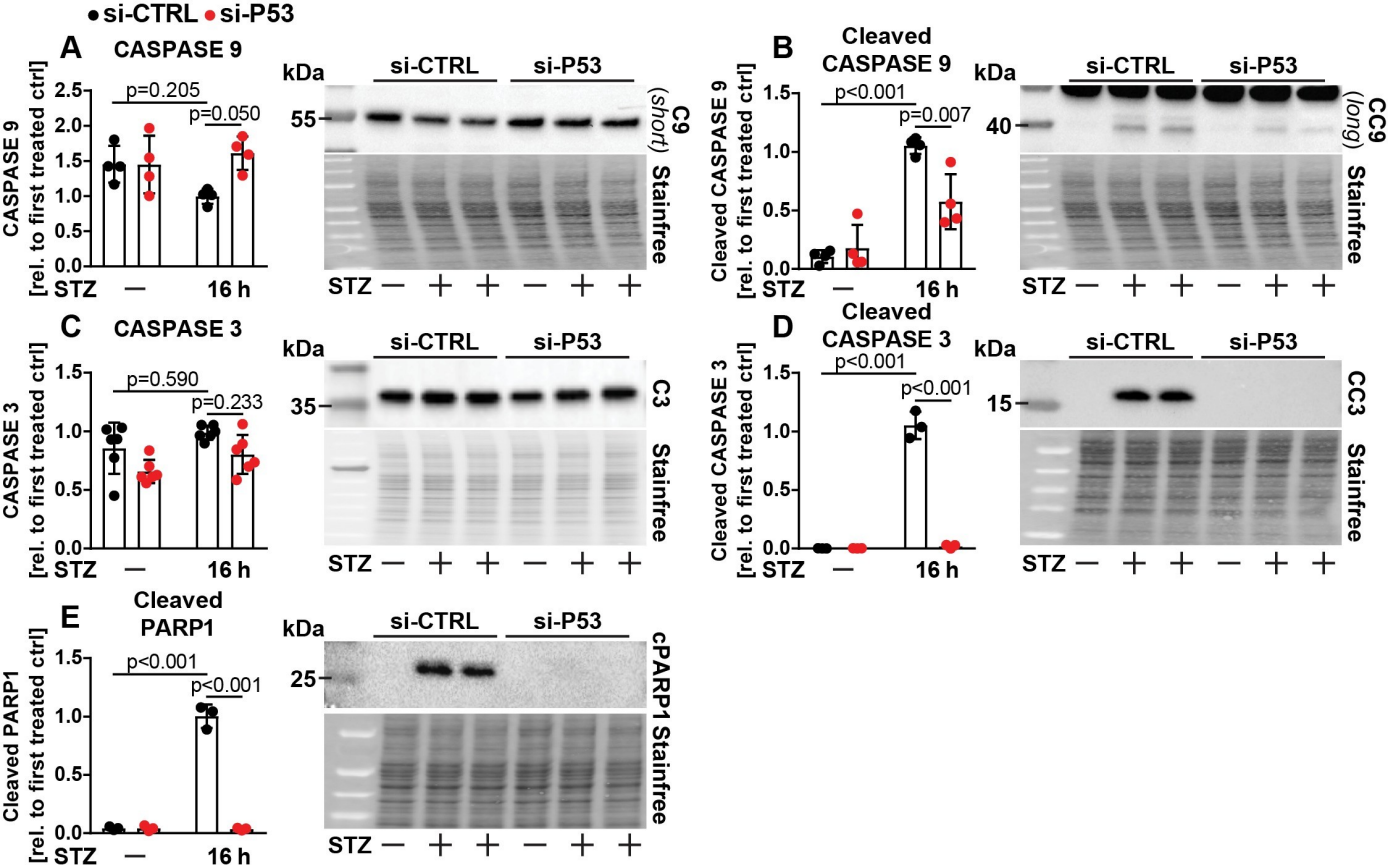
P53 activates STZ induced apoptotic signaling in Ins1E cells. Relative protein amount of (A) CASPASE 9 (C9), (B) cleaved CASPASE 9 (CC9), (C) CASPASE 3 (C3), (D) cleaved CASPASE 3 (CC3) and (E) cleaved PARP1 in Ins1E cells transfected with control siRNA or siRNA targeting P53. 48 h post transfection, cells were treated for 16 h with STZ or medium as control (n = 3-6 independent experiments and one representative immunoblot). The total protein content was used as loading control. The first treated control was set to 1. Significance was determined by two-way ANOVA followed by Sidak’s multiple comparison test.

To demonstrate that these processes have an impact on apoptosis, we assessed protein levels of CASPASE 3 and 9 (C3/C9) in its uncleaved (inactive) and cleaved (active) forms (Fig 2A–2D). C9 is activated by the intrinsic pathway of apoptosis, leading in turn to activation (cleavage) of C3, which acts as the executor caspase [35]. Knockdown of P53 ameliorated STZ induced activation of C9 and C3, but did either not affect (C3) or prevent STZ induced reduction (C9) of the uncleaved forms (Fig 2A–2D). In line with these findings, STZ treatment strongly elevated protein levels of cleaved PARP1, which is targeted by active caspases and a marker of ongoing apoptosis [42], which was almost completely abolished by P53 knockdown (Fig 2E). These data demonstrate that Ins1E beta cells are sensitive to STZ induced apoptosis in a P53 dependent manner [8], and reveal that P53 controls activation of P38 MAPK as well as C9 and C3 upon STZ treatment *in vitro*.

**Fig 3:**
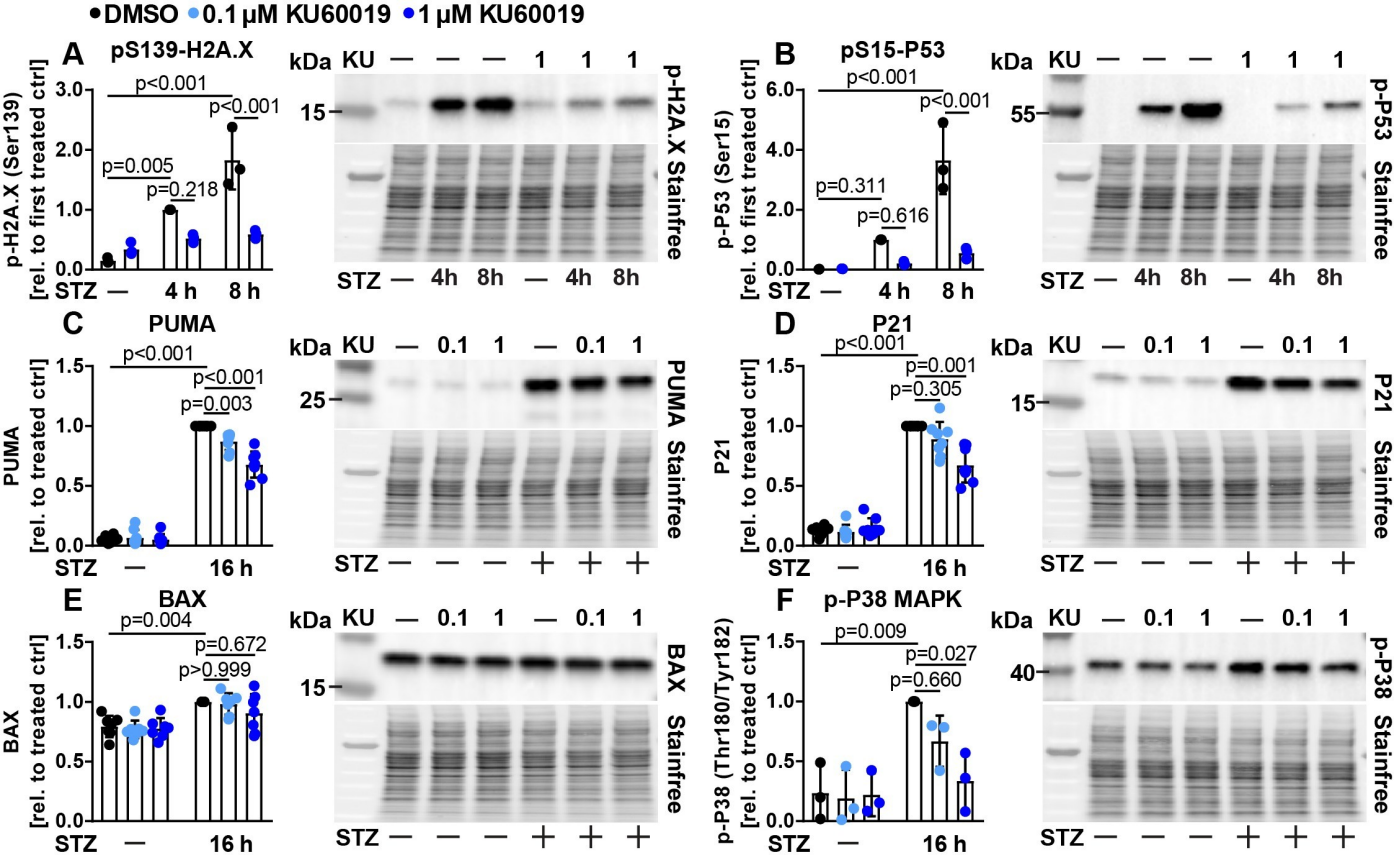
KU-60019 modulates STZ induced stress signaling pathways in Ins1E cells. Relative protein amount of (A) pS139-H2A.X, (B) pS15-P53, (C) PUMA, (D) P21, (E) BAX and (F) p-P38 MAPK in Ins1E cells treated for (A+B) 4 h or 8 h or (C-F) 16 h with 0.1 or 1 μM KU (or DMSO as control) and STZ or medium as control (n = 3–8 independent experiments and one representative immunoblot). The total protein content was used as loading control. The (first) treated control was set to 1. Significance was determined by two-way ANOVA followed by Sidak’s multiple comparison test.

Next, we aimed to investigate if a reduction of ATM activity would also ameliorate STZ induced cell signaling and death. Knockdown efficiency of several siRNAs targeting ATM was lower compared to knockdown achieved by the P53 siRNA (most effective ATM siRNA approx. 50% vs. P53 siRNA 85% as determined by qPCR, S1A Fig and S3A Fig). Attempts to detect rat ATM protein in Ins1E cell lysates failed due to inadequate antibody specificity and sensitivity (data not shown). To demonstrate the efficacy of the knockdown, we therefore used an antibody that recognizes proteins that are phosphorylated by ATM (or the related kinase Ataxia Telangiectasia and Rad3 Related, ATR). Knockdown of ATM partially reduced the cellular content of phosphorylated ATM/ATR target proteins (S3B Fig), and resulted in a significant reduction in STZ induced cell death (S3C Fig). In a second approach, we used the established second generation ATM inhibitor KU-60019 (hence denoted as KU) [43] in additional experiments. KU treatment also reduced the abundance of ATM/ATR phosphorylated proteins (S3D Fig), and dose dependently was able to inhibit STZ mediated cell death as determined by propidium iodide (PI) staining and flow cytometry (S3E Fig). We next assessed cellular stress signaling events as performed for P53 knockdown before. ATM is known to be one of the kinases able to phosphorylate histone H2A.X upon DNA damage and indeed, ATM inhibition blunted phosphorylation of histone H2A.X at serine 139 in our model system after 8 h STZ treatment (Fig 3A). ATM is known to induce phosphorylation of S15-P53 upon DNA damage [44]. Inhibition of ATM with KU drastically abrogated phosphorylation of S15-P53, indicating that ATM plays a major role in the regulation of the phosphorylation of this residue upon DNA damage in beta cells *in vitro* (Fig 3B). In contrast, IĸBα was mildly upregulated after 4 h and similar between DMSO and KU treated samples after 8 h STZ treatment (S3F Fig). In line with ATM mediated activation of P53, KU treatment significantly reduced protein levels of PUMA and P21 as well as activation of P38 MAPK, whereas BAX expression was unchanged (Fig 3C–3F). Similar to P53 knockdown, KU treatment had no effect on PDX1 protein expression (S3G Fig). Overall, ATM inhibition mainly phenocopied the effects achieved by P53 knockdown (except for pS139-H2A.X expression), although the effect on P53 target proteins tended to be slightly weaker.

**Fig 4:**
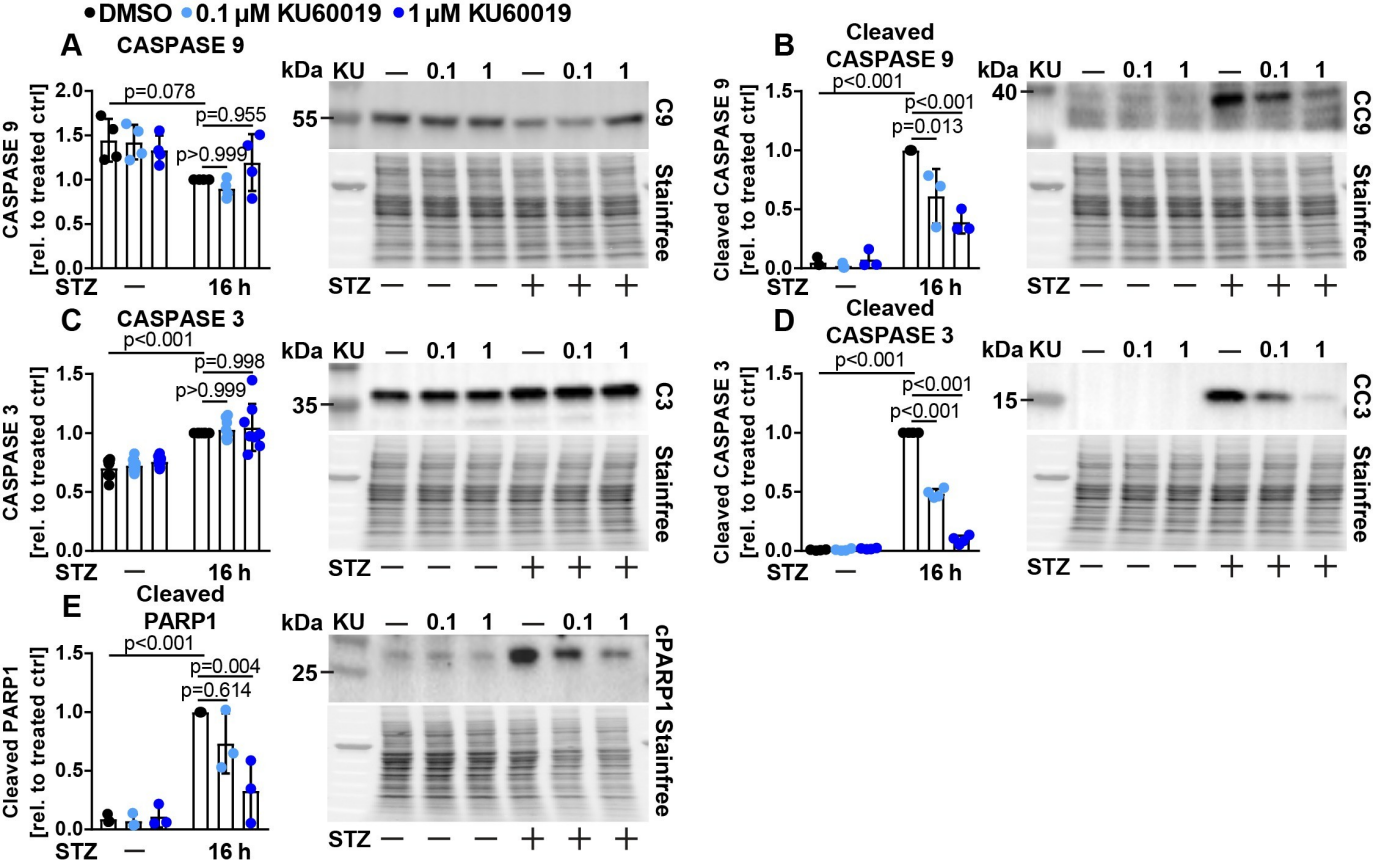
KU-60019 reduces STZ induced apoptotic signaling in Ins1E cells. Relative protein amount of (A) CASPASE 9 (C9), (B) cleaved CASPASE 9 (CC9), (C) CASPASE 3 (C3), (D) cleaved CASPASE 3 (CC3) and (E) cleaved PARP1 in Ins1E cells treated for 16 h with 0.1 or 1 μM KU (or DMSO as control) and STZ or medium as control (n = 3-8 independent experiments and one representative immunoblot). The total protein content was used as loading control. The treated control was set to 1. Significance was determined by two-way ANOVA followed by Sidak’s multiple comparison test.

We next assessed C9 and C3 signaling in KU treated Ins1E cells. STZ treatment again induced strong activation of both C9 and C3 and mildly decreased and increased uncleaved C9 and C3 protein levels, respectively (Fig 4A–4D). KU treatment dose dependently reduced activation of both caspases as well as cleaved PARP1, but did not alter levels of the uncleaved caspases (Fig 4A–4E). Overall, we demonstrate that ATM mediates phosphorylation of H2A.X and S15-P53, controls expression of P53 target genes such as PUMA and P21 and modulates STZ induced apoptosis signaling in beta cells.

### ATM and P53 differentially control beta cell survival upon inflammation, lipotoxicity and ER stress

**Fig 5:**
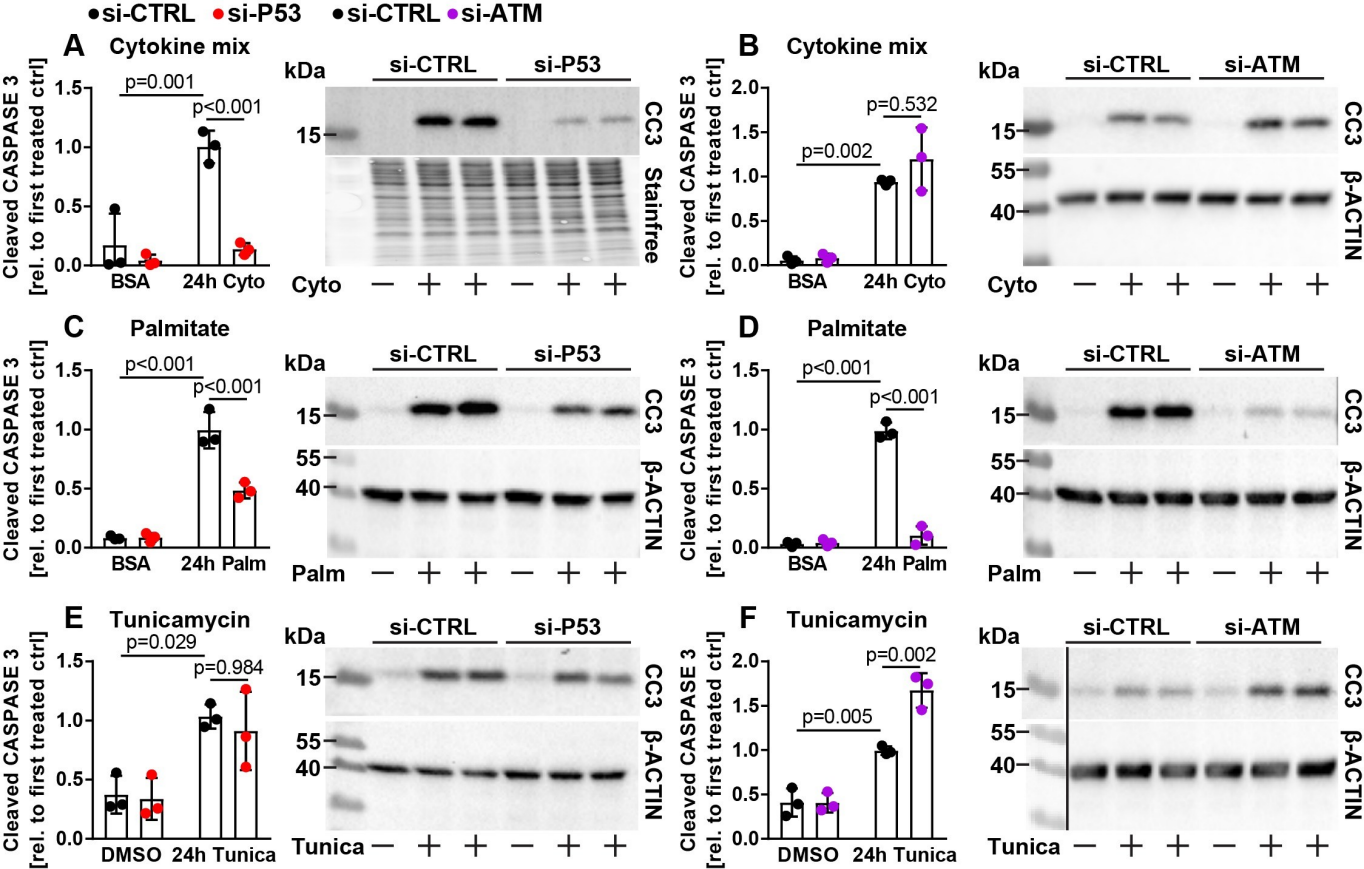
ATM and P53 differentially regulate beta cell apoptosis upon inflammation, lipotoxicity and ER stress. Relative protein amount of cleaved CASPASE 3 (CC3) in Ins1E cells transfected with control siRNA or siRNA targeting (A, C, E) P53 or (B, D, F) ATM. 24 h post transfection, cells were treated for 24 h with (A, B) the cytokine mix, (C, D) palmitate or (E, F) 2 μg/ml tunicamycin (n = 3 independent experiments and one representative immunoblot). The total protein content or beta ACTIN were used as loading control. The first treated control was set to 1. Significance was determined by two-way ANOVA followed by Sidak’s multiple comparison test. The black line in F indicates that the ladder was run on the same gel but not adjacent to samples depicted.

Several DNA damage independent mechanisms of beta cell death have been suggested to also occur in human beta cells during development of T2D, as well as in beta cells of T2D animal models [10, 15, 18, 27, 28, 45–49]. For this reason, we modelled inflammation (induced by combined action of tumor necrosis factor alpha (TNFα), interferon gamma (IFNγ) and interleukin 1 beta (IL1β)), lipotoxicity (by palmitate treatment) as well as ER stress (triggered by the chemical compound tunicamycin) using established experimental paradigms. The cytokines TNFα, IFNγ and IL1β synergistically induce beta cell death due to induction of inflammatory pathways such as NFĸB signaling, and hence simulate events potentially relevant to both T1D and T2D [17, 50]. Mechanistically, palmitate treatment is known to induce beta cell death in part due to generation of reactive oxygen species as well as increased synthesis of complex proapoptotic lipids such as ceramides [50–52]. Tunicamycin disturbs N-glycosylation of nascent proteins in the ER, causing misfolding and hence activates the ER stress response [15, 45, 46, 53]. We targeted ATM or P53 and exposed these cells to the cytokine mix, palmitate or tunicamycin. As expected, all treatments induced C3 cleavage in control siRNA treated cells (Fig 5A–5F). Strikingly, we found several differences between ATM and P53 knockdown cells. While knockdown of P53 strongly protected Ins1E cells against inflammation induced apoptosis, knockdown of ATM failed to do so (Fig 5A and 5B). In contrast, upon palmitate treatment, both ATM and P53 manipulation was sufficient to ameliorate cleavage of C3 (Fig 5C and 5D). While ER stress induced apoptosis was strikingly further increased by ATM knockdown, tunicamycin induced apoptosis was similar between control and P53 ablated Ins1E cells (Fig 5E and 5F). These results indicate that P53 and ATM differentially and depending on the cell stress stimulus, regulate apoptotic signaling in Ins1E cells.

Since ER stress appears to be highly relevant for beta cell demise and diabetes etiology [20, 27, 45, 46, 48, 54, 55], we further investigated the molecular mechanisms underlying the different outcome after manipulation of P53 (no regulation) and ATM (further increased cell death) in tunicamycin treated cells (Fig 5E and 5F). Two major pathways belonging to the unfolded protein response (UPR) are critical to sense and overcome ER stress in pancreatic beta cells. Accumulation of unfolded proteins leads to phosphorylation of the ER membrane resident kinase/endoribonuclease Inositol-requiring Enzyme 1 alpha (IRE1α), which upon activation induces splicing of a cryptic intron in the transcription factor X-Box Binding Protein (XBP1) mRNA, leading to generation of spliced XBP1 protein (XBP1s), which in turn stimulates expression of various chaperones to increase the folding capacity of the cell [47, 56, 57]. In parallel, an increase in unfolded proteins also leads to phosphorylation of the kinase Protein Kinase R-like Endoplasmic Reticulum Kinase (PERK, also known as EIF2AK3), which activates a signaling cascade culminating in an increased expression of the transcription factor Activating Transcription Factor 4 (ATF4), which acts to protect beta cells from ER stress associated death [56, 58]. We assessed acute activation of these pathways after short term treatment (6 h) with tunicamycin in combination with either P53 or ATM manipulation. At the time point and concentration tested, tunicamycin treatment induced XBP1s, but failed to significantly increase phosphorylation of IRE1α, while ATF4 protein was not detectable (S4A Fig, S4B Fig and data not shown). P53 or ATM manipulation did not affect these findings significantly (S4A Fig, S4B Fig). We therefore used a second inducer of ER stress, the small molecule thapsigargin, which disturbs calcium handling in the ER and therefore induces ER stress [59, 60]. In line with our experience that thapsigargin is more potent than tunicamycin at commonly used concentrations in Ins1E cells, thapsigargin significantly induced phosphorylation of IRE1α, splicing of XBP1 and protein levels of ATF4 (S4C Fig–S4E Fig). Nonetheless, activation of these pathways was not consistently different upon P53 and ATM manipulation, since only ATM knockdown but not ATM inhibition mildly increased XBP1s and ATF4 levels compared to control and P53 knockdown (S4D Fig and S4E Fig). Our results indicate that ATM dependent, P53 independent regulation of Ins1E survival after ER stress may not be mediated through these pathways.

### Quantitative comparison of the roles of ATM and P53 in Ins1E cell survival

**Fig 6:**
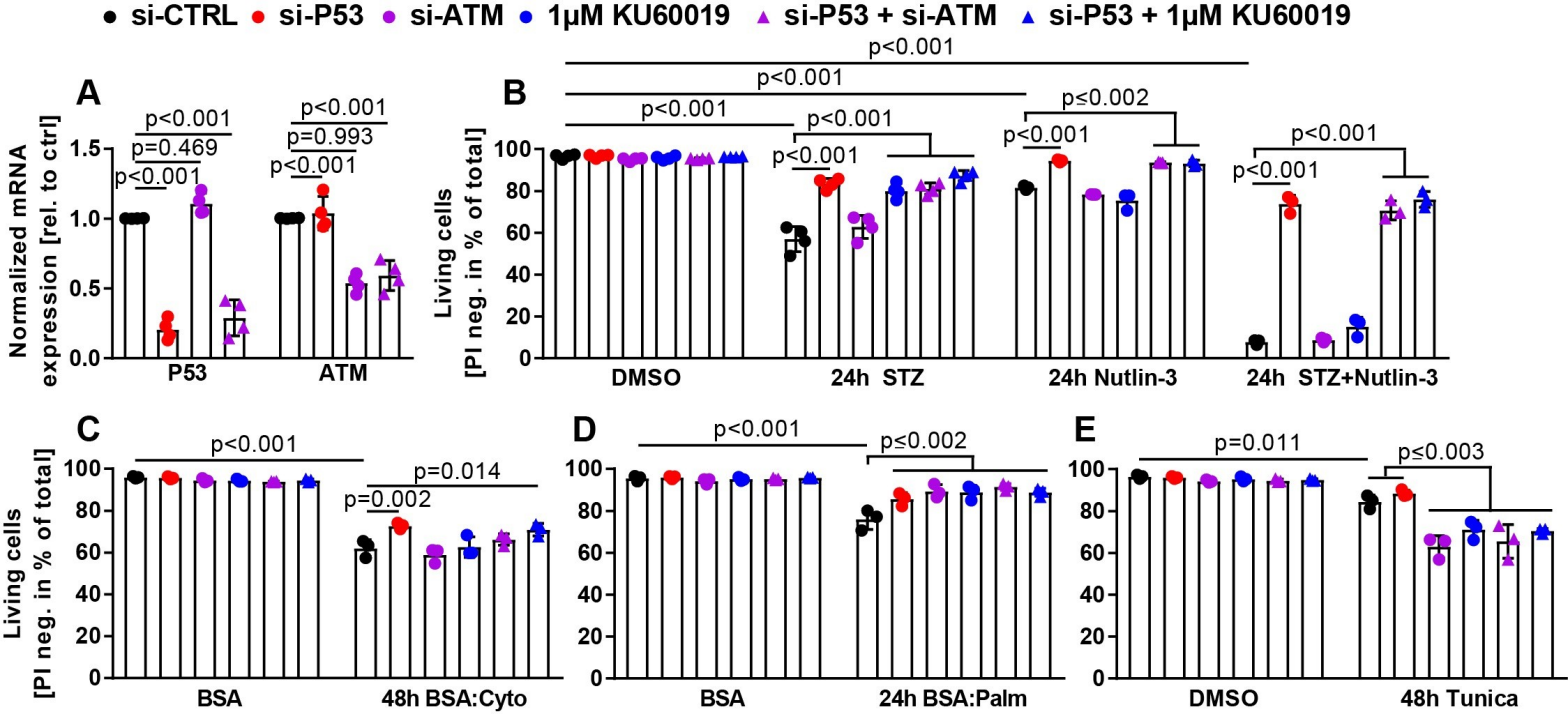
ATM and P53 differentially regulate beta cell death upon multiple types of cell stress. (A) Relative mRNA expression of *P53* and *ATM* in Ins1E cells 48 h post transfection normalized to the housekeeping genes *36B4* and *GUSB* (n = 4 independent experiments). (B-E) Flow cytometric Live/Dead analysis of Ins1E cells treated with control siRNA, siRNA targeting P53 or ATM, and 1 μM KU (either in combination or in single treatments as stated in the figure). 24 h post transfection, cells were treated for (B) 24 h with STZ, Nutlin-3 or STZ+Nutlin-3, (C) 48 h with the cytokine mix, (D) 24 h with palmitate or (E) 48 h with 6 μg/ml tunicamycin. Unstressed controls were treated with DMSO or BSA as indicated. Percentage of living cells was quantified using propidium iodide as viability stain (PI positive: dead; PI negative: alive) (n = 3–4 independent experiments). Significance was determined by (A) one-way or (B-E) two-way ANOVA followed by Sidak’s multiple comparison test.

While our data clearly indicate that P53 and ATM can regulate apoptotic processes (such as cleavage of caspases), other types of beta cell death such as necrosis might also occur and (potentially at the same time) be regulated by ATM and P53 signaling [33, 61, 62]. Therefore, we set out to directly quantify cell death as determined by irreversible failure of Ins1E cell membrane integrity. Moreover, we aimed to investigate if P53 and ATM manipulation would act additively, synergistically or co-dependent in our model system. We treated Ins1E cells side by side with si-P53, si-ATM, KU, a combination of si-P53+si-ATM or si-P53+KU and accordingly with STZ, Nutlin-3, STZ+Nutlin-3 (simulating maximal stimulation of DNA damage and P53 mediated cell death), palmitate, the cytokine mix and tunicamycin (as well as all relevant siRNA, DMSO and BSA controls). For this flow cytometry experiment, treatment time and concentrations were adapted to allow significant levels of cell death (see methods). We again confirmed knockdown efficiency of the siRNAs transfected alone or in combination in this experimental approach (Fig 6A). Consistently, neither P53 or ATM knockdown nor KU treatment affected basal levels of Ins1E cell survival, while all six treatments inducing cell stress resulted in significantly reduced cell survival compared to the respective diluent controls (DMSO or BSA, Fig 6B–6E). STZ, Nutlin-3 as well as STZ+Nutlin-3 treatments reduced cell viability by approx. 40%, 15% and 89%, respectively (Fig 6B). Importantly, and in line with our results on caspase activity, P53 knockdown greatly reduced cell death in each case (Fig 6B). ATM inhibition or knockdown increased survival after STZ treatment, but failed to do so upon Nutlin-3 or STZ+Nutlin-3 treatment, in line with the notion that Nutlin-3 activates P53 independently of ATM (Fig 6B). Combining ATM and P53 manipulation could not further increase cell survival (Fig 6B). Interestingly, both ATM knockdown and inhibition tended to mildly increase cell death upon Nutlin-3 treatment (Fig 6B). In line with our findings on caspase activation, P53 knockdown but not ATM manipulation significantly increased Ins1E cell survival after cytokine mix treatment (Fig 6C). Both P53 and ATM manipulation prevented cell death after palmitate treatment (Fig 6D). Finally, cell death upon tunicamycin treatment was significantly and further increased by ATM manipulation, but not significantly affected by P53 knockdown (Fig 6E), consistent with C3 activation demonstrated before (Fig 5E and 5F). These data demonstrate that ATM and P53 differentially control Ins1E cell survival as determined by quantifying loss of membrane integrity.

## Discussion

Several lines of evidence point to a crucial role of P53 in beta cell survival and diabetes. P53 was found to mediate beta cell death in patients (and mice) caused by a rare mutation in the glucokinase gene [16]. Palmitate induced cell death was linked to P53, inhibition of growth signaling and the protein p66(Shc) [63, 64]. On the other hand, conflicting reports indicated that P53 is not relevant for beta cell apoptosis (but sometimes instead regulates other cellular processes, such as mitophagy), taking into account that several of these studies were performed with the help of conventional whole body P53 knockout mice, which suffer from spontaneous, early onset cancer [65–67]. An additional report suggested that pharmacological systemic P53 activation can even ameliorate chemically induced diabetes [40], while ablation of one key inhibitor of P53 (MDM2) resulted in reduced insulin secretion without any effect on beta cell mass [68]. Based on these different studies, we conclude that the role of P53 in beta cell demise is controversial, likely at least in part due to different models and experimental setups being tested. The results presented here demonstrate a complex and striking role for the DDR proteins P53 and its upstream regulator ATM in beta cell survival upon diverse types of diabetogenic beta cell stress *in vitro*. Importantly, we demonstrate that in our experimental setting, both ATM and P53 modulate the activation of the intrinsic pathway of apoptosis in beta cells and the stress kinase P38 MAPK upon STZ treatment (Figs 1–4). While several paralogues of P38 exist, P38δ has been previously linked to beta cell survival [69]. Furthermore, inhibition of ATM also strongly reduces phosphorylation of its known target protein histone H2A.X, which is broadly used as an indicator of DNA damage. This finding demonstrates that ATM is necessary for full STZ treatment induced phosphorylation of this histone in Ins1E cells (Fig 3A), and future experiments should use ATM independent markers to demonstrate DNA damage when manipulating ATM. Further, ablation of P53 protects against STZ induced beta cell death, which could not be further enhanced by ATM knockdown or inhibition (Fig 6B). Since STZ uptake is selectively dependent on GLUT2 expression [38, 70], and STZ induced DNA damage appears not to be affected by P53 knockdown as determined by similar phosphorylation of H2A.X (Fig 1A), we also conclude that manipulation of P53 does not impair STZ uptake into the Ins1E cells nor influence the occurrence of DNA damage itself. Furthermore, we demonstrate that ablation of either ATM or P53 diminishes STZ induced activation of C9 and C3 without reducing uncleaved C9 and C3, demonstrating that STZ induces the intrinsic pathway of apoptosis dependent on both proteins *in vitro* (Figs 2 and 4). STZ treatment of cells and in animal models is widely used to study diabetes, but is inconsistently presented as a model for T1D or T2D [8, 37, 71]. As others have reported that STZ is able to induce hyperglycemia to the same extent in wildtype mice as in immunodeficient mice [72], and STZ can induce cell death in beta cell lines without presence of immune cells (e.g. this study), we caution that experimental STZ treatments may not primarily model cellular processes relevant to T1D. Indeed, high fat feeding as a model for T2D development has been shown to induce DNA damage in pancreatic beta cells [73], therefore STZ treatment may model at least some of the mechanisms relevant for T2D etiology.

Strikingly, pharmacological systemic activation of P53 by Nutlin-3 was shown to ameliorate STZ induced diabetes in mice [40]. In light of our data regarding the effect of P53 in beta cells, we posit that the observed protection against diabetes after systemic Nutlin-3 treatment is mediated by P53 in non-beta cells [40], although differences in beta cell biology between rats and mice may also be relevant here. Our mechanistical analyses reveal for the first time that while ablation of either ATM or P53 significantly and drastically protect against STZ induced beta cell death, inhibition of ATM cannot further add to the protection induced by P53 knockdown *in vitro* (Fig 6B). This finding combined with direct evidence for ATM regulation of the key residue Ser15 of P53 indicates that STZ induced activation of P53 by ATM is a critical event in the context of DNA damage in beta cells *in vitro*.

Furthermore, we directly assess and compare the ability of ATM and P53 to affect beta cell apoptosis induced by three additional diabetogenic cellular insults in addition to STZ, allowing us to compare the magnitude of protection granted by ATM or P53 ablation against each insult, which has not been done before to our knowledge. Although a protective effect of P53 ablation was already demonstrated for example in the context of lipotoxicity *in vitro* [63], the effective protection against each of the four important, and on a molecular level very diverse insults (DNA damage, inflammation, lipotoxicity, ER stress), has not been compared. We note that while knockdown of P53 significantly protects against three of four types of stress, protection against DNA damage induced apoptosis is greatest, followed by inflammation, lipotoxicity, and with protection against ER stress not reaching significance (Figs 5 and 6). This aligns with the notion that DNA damage is a very strong activator of P53. Moreover, DNA damage induced activation of the intrinsic pathway of apoptosis including activation of C9 and Cytochrome C release is known to be controlled at multiple levels by P53 (which is activated by ATM through S15-P53 phosphorylation), in part through increased expression of canonical P53 target genes such as *PUMA* [74, 75] and localization of BAX [76]. Indeed, the role of P53 to ensure DNA stability is conserved also in more primitive and evolutionary older species [77]. The effect of P53 knockdown on inflammatory stress was smaller compared to DNA damage. We suppose that cytokines mainly activate P53 independent inflammatory signaling, although it is clear that cytokine treatment also induces DNA damage in human islets [78]. Finally, we speculate that activation of P53 is weaker upon lipotoxicity and ER stress compared to DNA damage, leading to only a small impact of P53 manipulation on C3 activation and cell death. We note that this is potentially cell type specific [79].

Furthermore, we demonstrate that ATM action differentially, and dependent on the type of stress, can increase or decrease beta cell death. Hence, ATM inhibition offered protection against STZ induced apoptosis (although weaker compared to P53 knockdown), in line with its known function in the DDR [44]. Protection against lipotoxicity tended to be stronger upon ATM manipulation compared to P53 knockdown. Analysis of the lipotoxic response in ATM and/or P53 null beta cells would be helpful to clarify this finding. Notably, ATM manipulation failed to significantly alter inflammation associated apoptosis while P53 knockdown was protective (Figs 5A, 5B and 6C). Hence, during inflammatory beta cell stress, ATR or a related kinase could be more important than ATM for P53 activation [80]. Notably, tunicamycin induced ER stress associated cell death was massively and significantly increased, which was not prevented by P53 manipulation (Figs 5E, 5F and 6E). These findings underscore the important but complex roles of both DDR proteins for beta cell survival *in vitro*. We demonstrate that while both proteins likely act in the same pathway in STZ induced apoptosis, ATM controls additional, P53 independent pathways especially upon ER stress. In other cell types, ATM has been linked to regulation of IRE1α, which is critical for the cellular ER stress response [81]. Nonetheless, we found no clear evidence for strongly altered IRE1 activation, XBP1 splicing or ATF4 induction after short term tunicamycin or thapsigargin treatment (S4 Fig). Since P53 ablation does not affect the ATM manipulation induced increase in ER stress stimulated cell death, we conclude that P53 independent, ATM dependent targets control tunicamycin induced ER stress in Ins1E cells. In addition, we fail to detect any protective effect of ATM ablation upon inflammatory cell stress, while P53 knockdown is protective. We hypothesize that P53 is activated by non-ATM signaling in this context. Our findings underscore that signaling of different DDR proteins can lead to opposite outcomes, hence ATM negatively affects cell survival upon DNA damage, but increases cell survival upon ER stress. Analysing the phosphoproteome of beta cells with ablation or inhibition of ATM (and optimally, ATR) will help to fully understand the molecular processes and identify the target proteins phosphorylated and regulated by this critical kinase, for example during ER stress. Of interest, ATR has been indirectly linked to beta cell proliferation, suggesting that investigations into additional DDR proteins might further help to understand beta cell physiology on a molecular level [23, 82]. In detail, ATM manipulation cannot reproduce the same protective effect detected upon P53 knockdown after STZ and cytokine treatment, which might suggest that ATR may also play a role in beta cell survival, since it also is known to regulate phosphorylation of H2A.X and S15-P53 [44]. Since some monogenetic forms of diabetes such as MODY4 also appear to involve apoptotic processes mediated by P53 target genes [83], analyses of DDR activation in MODY patient samples or animal models could further help to understand these rare diseases. In addition, P53 has also been linked to beta cell apoptosis induced by additional mechanisms including hyperglycemia [84] as well as advanced glycation end products [85], and it appears worthwhile to analyze the impact of ATM under these conditions. As a general limitation of our study, our findings are based on acute genetic and pharmacological (but not Cre/loxP or CrispR/Cas9 mediated) manipulation of an immortal rat beta cell line, which shows lower inducibility of glucose stimulated insulin secretion compared to primary beta cells, likely as a compensation for its very high proliferation rate [86]. Further studies will be necessary to investigate the impact of the DDR on survival of beta cells in isolated islets and/or primary beta cells from mice, rats and humans (optimally with inducible, genetic ablation of DDR proteins). These experiments will help to evaluate whether manipulation of specific DDR proteins may have implications for improvement of beta cell survival during transplantation of islets, more efficient generation of stem cell derived beta cells, as well as treatment of diabetes.

## Conclusion

We demonstrate that ablation of both ATM and P53 protects against beta cell apoptosis induced by DNA damage and lipotoxicity in Ins1E cells. Combination experiments demonstrate that ATM inhibition fails to further ameliorate DNA damage induced beta cell apoptosis, when P53 is knocked down at the same time. In contrast, ATM manipulation fails to alter inflammatory beta cell death, and even increases ER stress induced apoptosis, while only P53 knockdown is protective upon cytokine treatment. We conclude that ATM and P53 differentially regulate beta cell death depending on the diabetogenic insult in Ins1E cells.

## Supporting information

S1 Fig P53 regulates proliferation and survival in Ins1E cells. (A-E) Relative mRNA expression levels of (A) *P53*, (B) *MDM2*, (C) *MDM4*, (D) *PHLDA3* and (E) *PDX1* in Ins1E cells transfected with control siRNA or siRNA targeting P53 and treated for 24 h with Nutlin-3 (or DMSO as control) 24 h post transfection, normalized to the housekeeping genes *36B4* and *GUSB* (n = 3 independent experiments). (F) Protein levels of pS15-P53 and cleaved CASPASE 3 (CC3) of Ins1E cells treated as in A-E (n = 3 independent experiments, showing one representative immunoblot). Alpha TUBULIN was used as loading control. (G) Automated cell counts of Ins1E cells 48 h after transfection with control siRNA or siRNA targeting P53 (n = 3 independent experiments, paired t-test p = 0.011). (H) Flow cytometric EdU analysis of Ins1E cells 48 h after transfection with control siRNA or siRNA targeting P53. EdU was added for 2.5 h before analysis (n = 3 independent experiments). Significance was determined by (A-E) two-way ANOVA followed by Sidak’s multiple comparison test or (G+H) by an unpaired, two-sided Student’s t-test.
(TIF)

S2 Fig P53 regulates STZ induced apoptotic signaling in Ins1E cells. (A-E) Relative mRNA expression levels of (A) *P53*, (B) *MDM2*, (C) *MDM4*, (D) *PHLDA3* and (E) *PDX1* in Ins1E cells transfected with control siRNA or siRNA targeting P53 and treated for 6 h with STZ or medium as control 42 h post transfection, normalized to the housekeeping genes *36B4* and *GUSB* (n = 4 independent experiments). (F+G) Relative protein amount of (F) IκBα and (G) PDX1 of Ins1E cells transfected with control siRNA or siRNA targeting P53. 48 h post transfection, cells were treated for (F) 4 h or 8 h or (G) 16 h with STZ or medium as control (n = 3–4 independent experiments and one representative immunoblot). The total protein content was used as loading control. The first treated control was set to 1. (A-G) Significance was determined by two-way ANOVA followed by Sidak’s multiple comparison test.
(TIF)

S3 Fig Additional data for ATM manipulation. (A) Relative mRNA expression levels of *ATM* in Ins1E cells transfected with control siRNA or siRNA targeting ATM 48 h post transfection, normalized to the housekeeping gene *36B4* (n = 3 independent experiments). (B+C) Relative protein amount of (B) pS/pT-ATM/ATR substrates and (C) cleaved CASPASE 3 (CC3) of Ins1E cells transfected with control siRNA or siRNA targeting ATM. Cells were treated for the final 16 h with STZ or medium as control (n = 3 independent experiments and one representative immunoblot). (D) Relative protein amount of pS/pT-ATM/ATR substrates of Ins1E cells treated for 16 h with 0.1 or 1 μM KU (or DMSO as control) and STZ or medium as control (n = 6 independent experiments and one representative immunoblot). (E) Flow cytometric Live/Dead analysis of Ins1E cells treated with 0.1, 0.2, 0.4, 0.8 or 1 μM KU (or DMSO as control) and STZ or medium as control. Percentage of living cells was quantified using propidium iodide as viability stain (PI positive: dead; PI negative: alive) (n = 3 independent experiments). (F+G) Relative protein amount of (F) IκBα and (G) PDX1 of Ins1E cells treated with 0.1 or 1 μM KU (or DMSO as control) and for (F) 4 h and 8 h or (G) 16 h with STZ or medium as control (n = 3–4 independent experiments and one representative immunoblot). (B-D, F+G) The total protein content or beta ACTIN was used as loading control. The first treated control was set to 1. Significance was determined by (A) an unpaired two-sided Student’s t-test, (B-D, F+G) two-way or (E) one-way ANOVA followed by Sidak’s multiple comparison test.
(TIF)

S4 Fig UPR regulation by ATM and P53. Relative protein amount of (A) p-IRE1α, (B) XBP1s, (C) p-IRE1α, (D) XBP1s and (E) ATF4 in Ins1E cells transfected with control siRNA or siRNA targeting P53 or ATM, or treated with 1 μM KU (or DMSO as control). 24 h post transfection, cells were treated for 6 h with (A+B) 2 μg/ml tunicamycin, (C-E) 1 μM thapsigargin or DMSO as control (n = 3 independent experiments and one representative immunoblot). The total protein content was used as loading control. The first treated control was set to 1. Significance was determined by two-way ANOVA followed by Sidak’s multiple comparison test.
(TIF)

S5 Fig Uncropped blots. (TIF)

S1 Table qPCR primer sequences. (TIF)
