# Supplementary figures and images for: ATM and P53 differentially regulate pancreatic beta cell survival in Ins1E cells
Source: PLoS One. 2020 Aug 18;15(8):e0237669. doi: 10.1371/journal.pone.0237669 (PMC7437460; doi:10.1371/journal.pone.0237669)

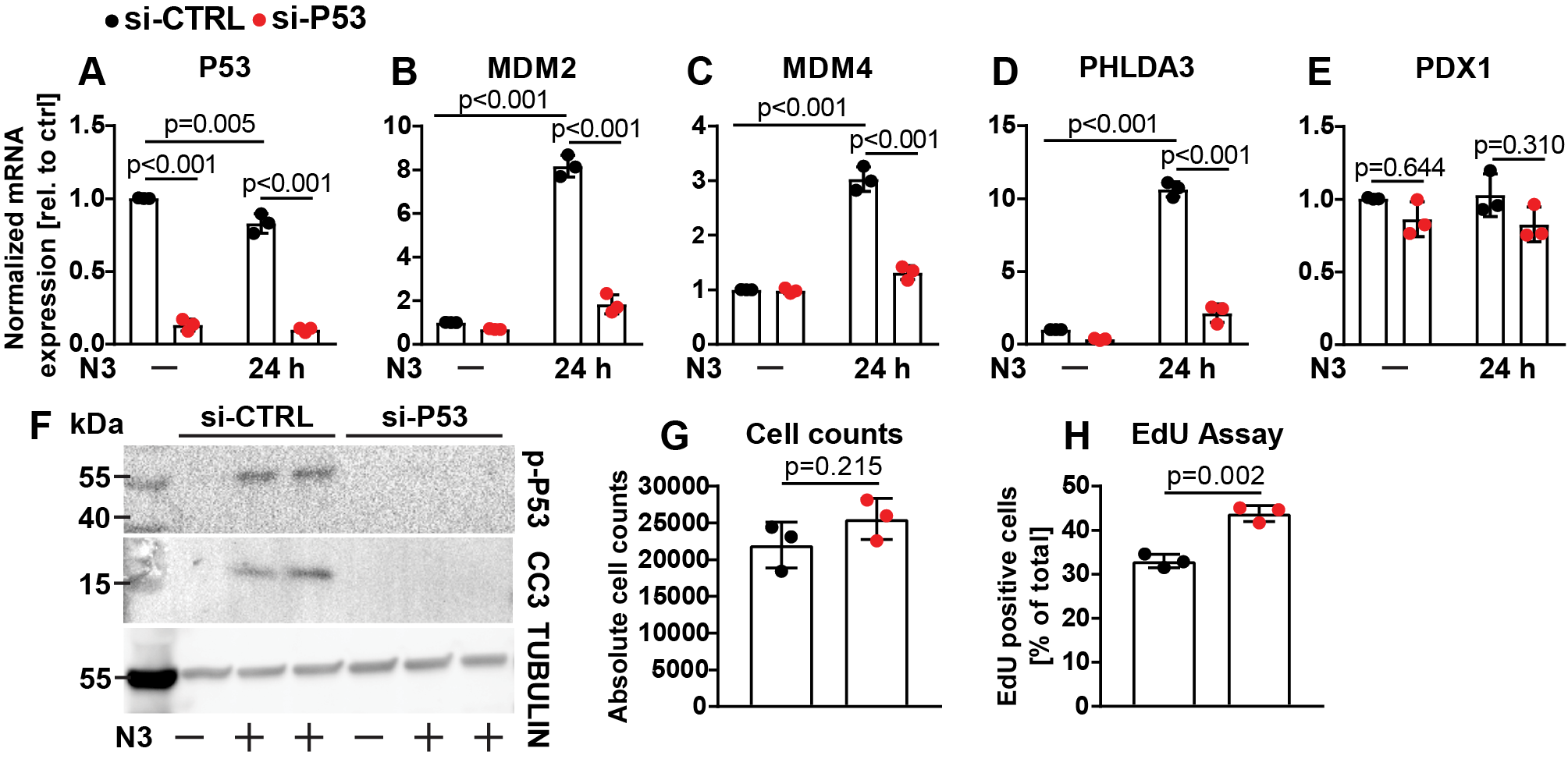

Supplement: S1 Fig — (A-E) Relative mRNA expression levels of (A) P53, (B) MDM2, (C) MDM4, (D) PHLDA3 and (E) PDX1 in Ins1E cells transfected with control siRNA or siRNA targeting P53 and treated for 24 h with Nutlin-3 (or DMSO as control) 24 h post transfection, normalized to the housekeeping genes 36B4 and GUSB (n = 3 independent experiments). (F) Protein levels of pS15-P53 and cleaved CASPASE 3 (CC3) of Ins1E cells treated as in A-E (n = 3 independent experiments, showing one representative immunoblot). Alpha TUBULIN was used as loading control. (G) Automated cell counts of Ins1E cells 48 h after transfection with control siRNA or siRNA targeting P53 (n = 3 independent experiments, paired t-test p = 0.011). (H) Flow cytometric EdU analysis of Ins1E cells 48 h after transfection with control siRNA or siRNA targeting P53. EdU was added for 2.5 h before analysis (n = 3 independent experiments). Significance was determined by (A-E) two-way ANOVA followed by Sidak’s multiple comparison test or (G+H) by an unpaired, two-sided Student’s t-test. (TIF) [file pone.0237669.s001.tif]

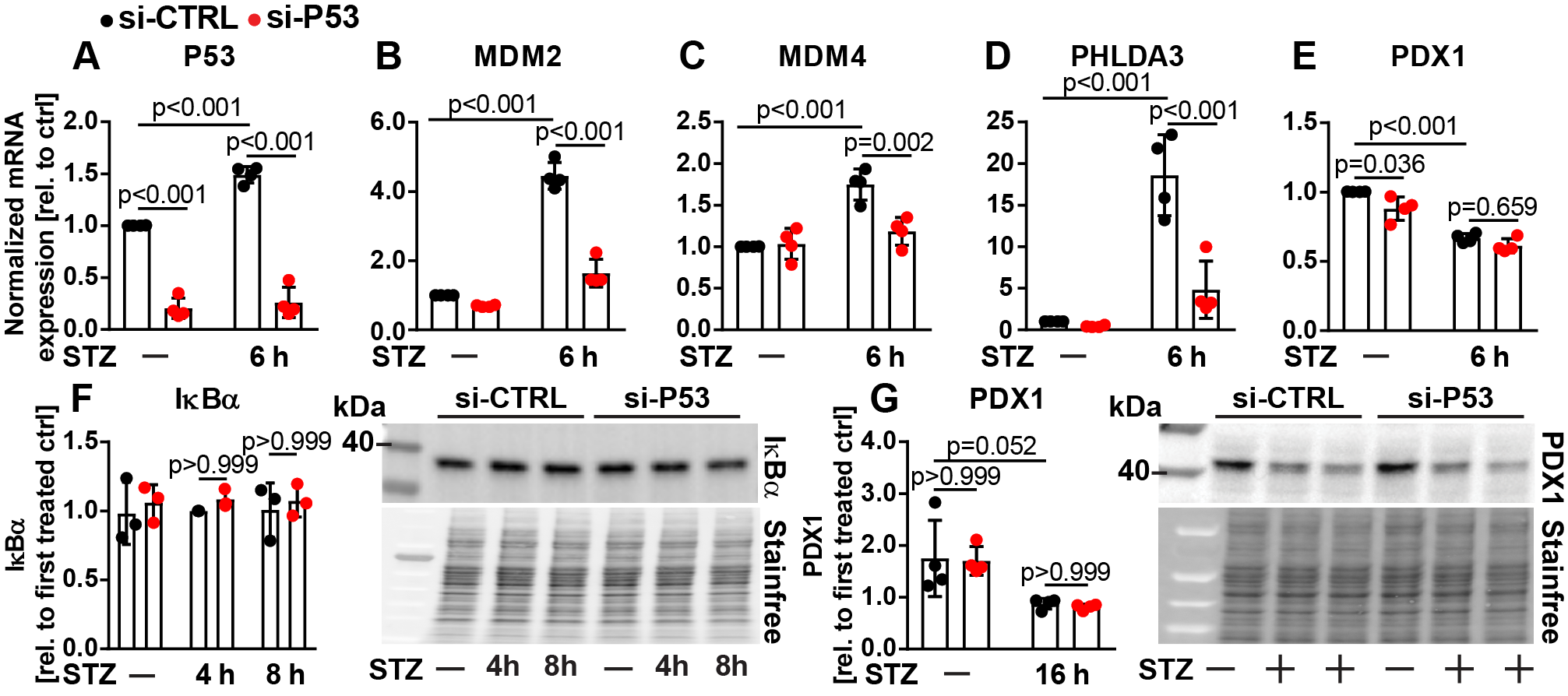

Supplement: S2 Fig — (A-E) Relative mRNA expression levels of (A) P53, (B) MDM2, (C) MDM4, (D) PHLDA3 and (E) PDX1 in Ins1E cells transfected with control siRNA or siRNA targeting P53 and treated for 6 h with STZ or medium as control 42 h post transfection, normalized to the housekeeping genes 36B4 and GUSB (n = 4 independent experiments). (F+G) Relative protein amount of (F) IκBα and (G) PDX1 of Ins1E cells transfected with control siRNA or siRNA targeting P53. 48 h post transfection, cells were treated for (F) 4 h or 8 h or (G) 16 h with STZ or medium as control (n = 3–4 independent experiments and one representative immunoblot). The total protein content was used as loading control. The first treated control was set to 1. (A-G) Significance was determined by two-way ANOVA followed by Sidak’s multiple comparison test. (TIF) [file pone.0237669.s002.tif]

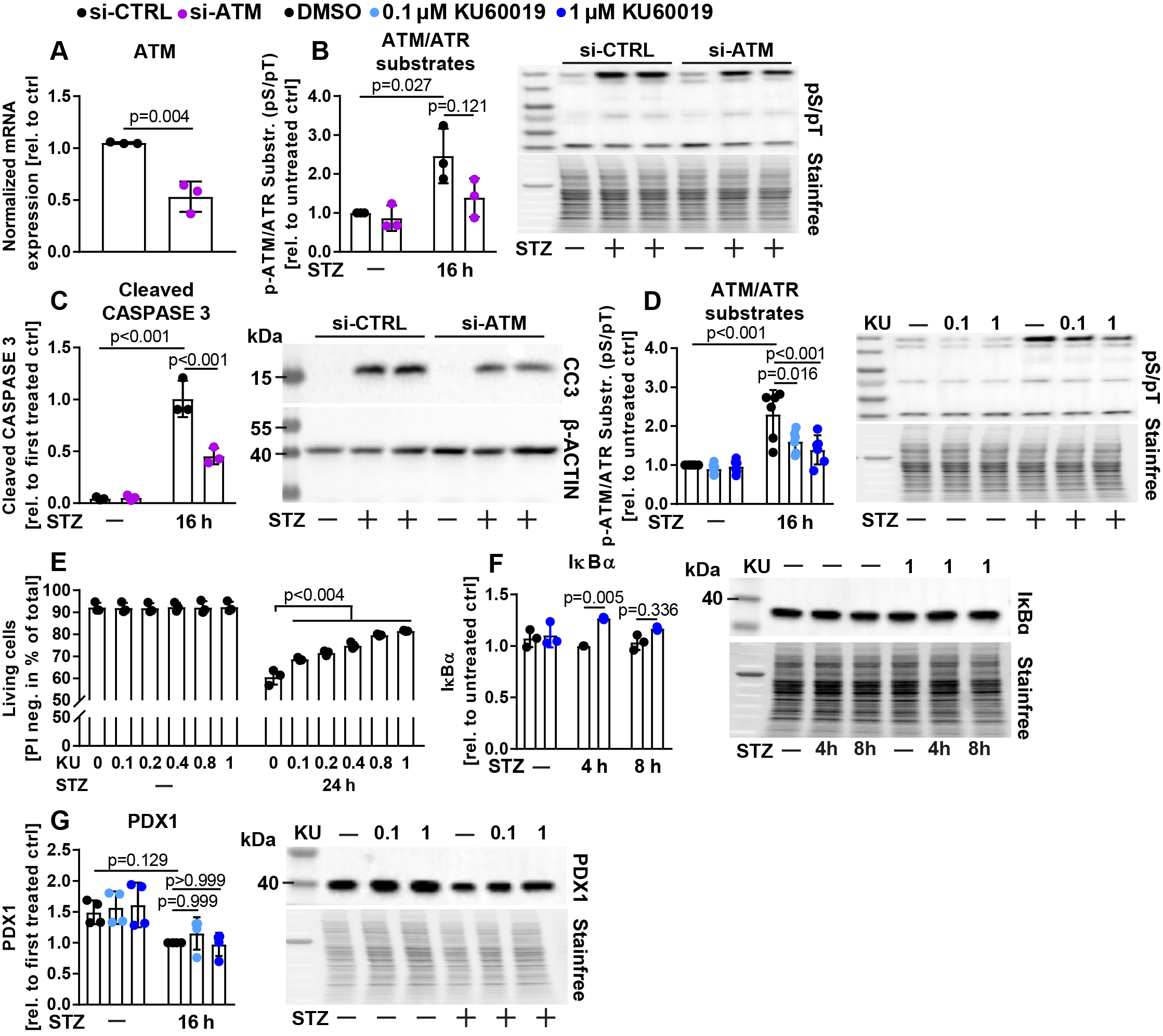

Supplement: S3 Fig — (A) Relative mRNA expression levels of ATM in Ins1E cells transfected with control siRNA or siRNA targeting ATM 48 h post transfection, normalized to the housekeeping gene 36B4 (n = 3 independent experiments). (B+C) Relative protein amount of (B) pS/pT-ATM/ATR substrates and (C) cleaved CASPASE 3 (CC3) of Ins1E cells transfected with control siRNA or siRNA targeting ATM. Cells were treated for the final 16 h with STZ or medium as control (n = 3 independent experiments and one representative immunoblot). (D) Relative protein amount of pS/pT-ATM/ATR substrates of Ins1E cells treated for 16 h with 0.1 or 1 μM KU (or DMSO as control) and STZ or medium as control (n = 6 independent experiments and one representative immunoblot). (E) Flow cytometric Live/Dead analysis of Ins1E cells treated with 0.1, 0.2, 0.4, 0.8 or 1 μM KU (or DMSO as control) and STZ or medium as control. Percentage of living cells was quantified using propidium iodide as viability stain (PI positive: dead; PI negative: alive) (n = 3 independent experiments). (F+G) Relative protein amount of (F) IκBα and (G) PDX1 of Ins1E cells treated with 0.1 or 1 μM KU (or DMSO as control) and for (F) 4 h and 8 h or (G) 16 h with STZ or medium as control (n = 3–4 independent experiments and one representative immunoblot). (B-D, F+G) The total protein content or beta ACTIN was used as loading control. The first treated control was set to 1. Significance was determined by (A) an unpaired two-sided Student’s t-test, (B-D, F+G) two-way or (E) one-way ANOVA followed by Sidak’s multiple comparison test. (TIF) [file pone.0237669.s003.tif]

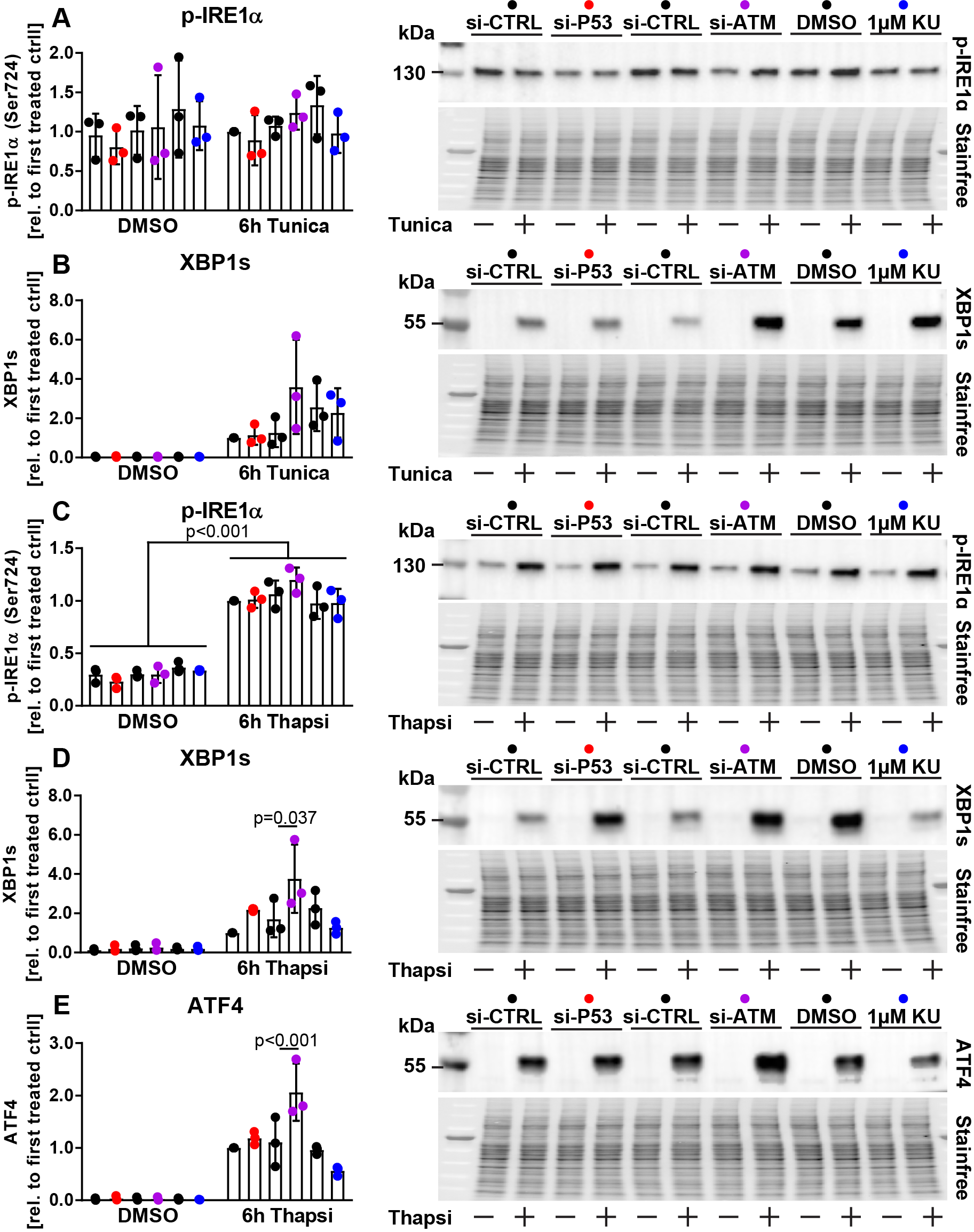

Supplement: S4 Fig — Relative protein amount of (A) p-IRE1α, (B) XBP1s, (C) p-IRE1α, (D) XBP1s and (E) ATF4 in Ins1E cells transfected with control siRNA or siRNA targeting P53 or ATM, or treated with 1 μM KU (or DMSO as control). 24 h post transfection, cells were treated for 6 h with (A+B) 2 μg/ml tunicamycin, (C-E) 1 μM thapsigargin or DMSO as control (n = 3 independent experiments and one representative immunoblot). The total protein content was used as loading control. The first treated control was set to 1. Significance was determined by two-way ANOVA followed by Sidak’s multiple comparison test. (TIF) [file pone.0237669.s004.tif]

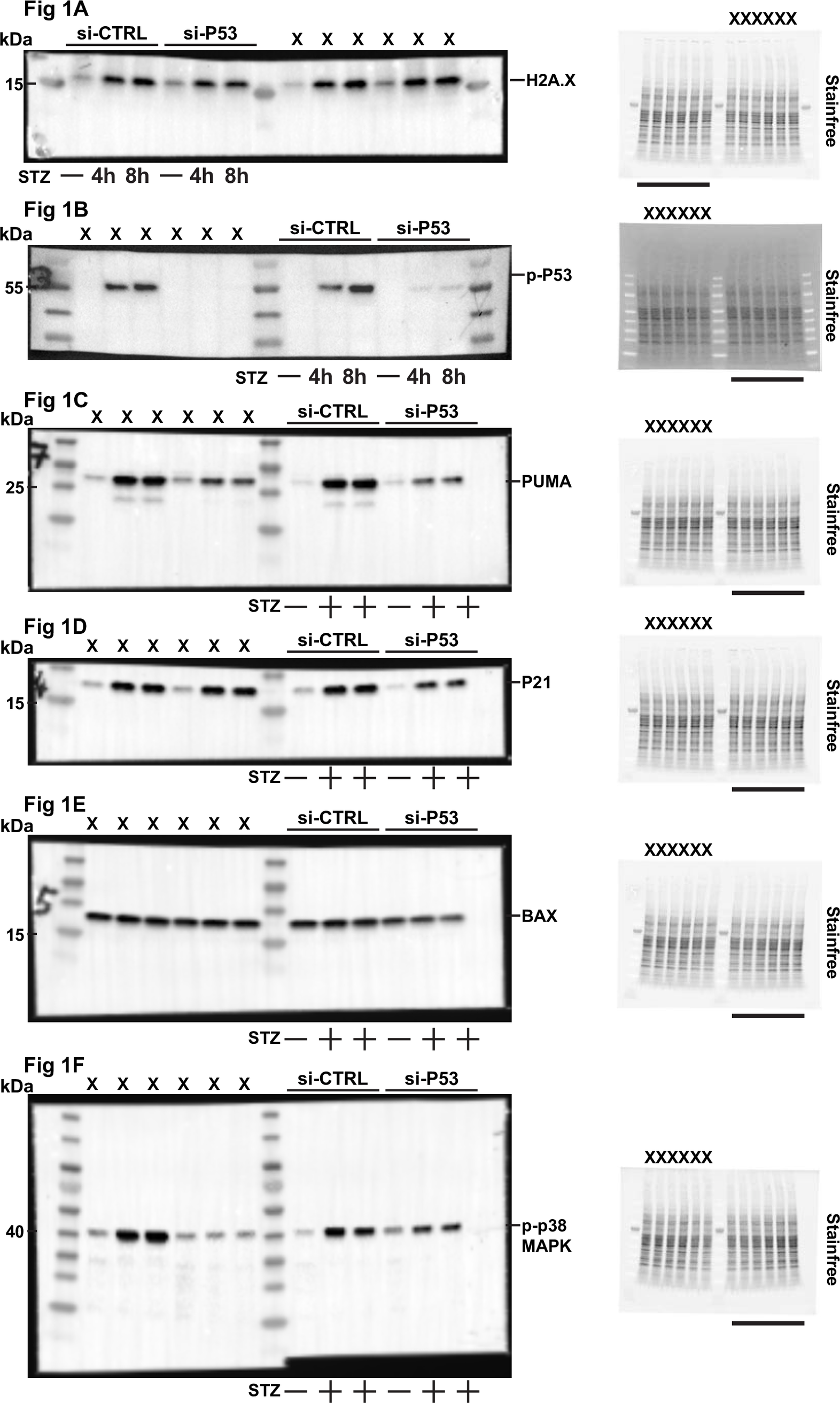

Supplement: S5 Fig — (TIF) [file pone.0237669.s005.tif]

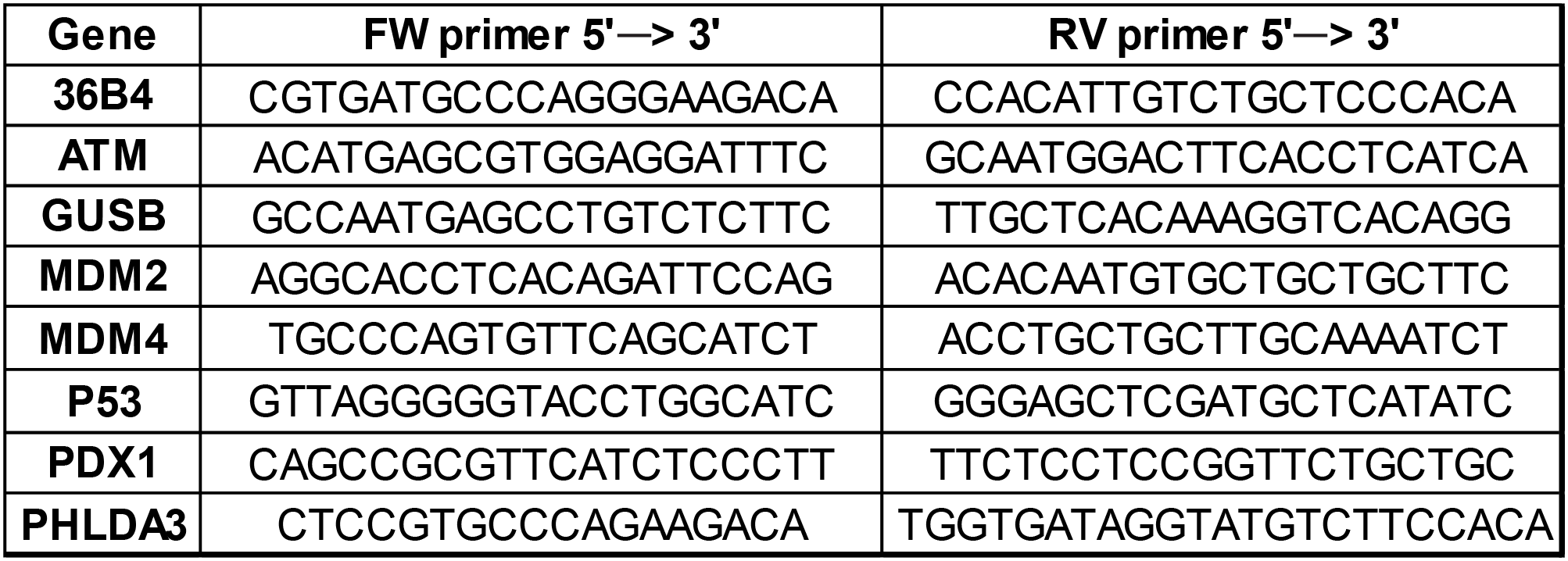

Supplement: S1 Table — (TIF) [file pone.0237669.s006.tif]
